# Online Prediction of Ship Behavior with Automatic Identification System Sensor Data Using Bidirectional Long Short-Term Memory Recurrent Neural Network

**DOI:** 10.3390/s18124211

**Published:** 2018-11-30

**Authors:** Miao Gao, Guoyou Shi, Shuang Li

**Affiliations:** 1 Navigation College, Dalian Maritime University, Dalian 116026, China; gaomiao4566@dlmu.edu.cn (M.G.); lishuang5997@dlmu.edu.cn (S.L.); 2 Collaborative Innovation Research Institute of Autonomous Ship, Dalian Maritime University, Dalian 116026, China; 3 Key Laboratory of Navigation Safety Guarantee of Liaoning Province, Dalian 116026, China

**Keywords:** ship behavior, machine learning, online prediction, AIS sensor data, big data

## Abstract

The real-time prediction of ship behavior plays an important role in navigation and intelligent collision avoidance systems. This study developed an online real-time ship behavior prediction model by constructing a bidirectional long short-term memory recurrent neural network (BI-LSTM-RNN) that is suitable for automatic identification system (AIS) date and time sequential characteristics, and for online parameter adjustment. The bidirectional structure enhanced the relevance between historical and future data, thus improving the prediction accuracy. Through the “forget gate” of the long short-term memory (LSTM) unit, the common behavioral patterns were remembered and unique behaviors were forgotten, improving the universality of the model. The BI-LSTM-RNN was trained using 2015 AIS data from Tianjin Port waters. The results indicate that the BI-LSTM-RNN effectively predicted the navigational behaviors of ships. This study contributes significantly to the increased efficiency and safety of sea operations. The proposed method could potentially be applied as the predictive foundation for various intelligent systems, including intelligent collision avoidance, vessel route planning, operational efficiency estimation, and anomaly detection systems.

## 1. Introduction

For a ship to avoid collision, it must predict the behaviors of other ships in order to estimate the collision risk. High-precision real-time prediction of ships’ navigational behaviors can effectively increase the reliability of collision avoidance decisions and decrease the risk of collisions.

Navigation behavior prediction algorithms can be divided into two categories: offline and online prediction. Numerous offline prediction algorithms are employed in the fields of trajectory estimation and data restoration. In offline prediction, trajectory data are input to a fixed formula or trained model. These algorithms are insufficiently flexible for adaptation to actual predicted data; they also lack timeliness and high efficiency. 

Examples of offline prediction studies include work by Han et al. [1] on the prediction of a ship’s trajectory by establishing a state-switching model; they proposed a conflict-free four-dimensional aircraft prediction method based on hybrid system theory. For predicting ship positions, Xu et al. [2] trained a three-layer back-propagation (BP) neural network to accept the ship’s direction and speed as inputs and yield differences in the latitude and longitude as output. Xu et al. [3] used a Kalman filter to modify automatic identification system (AIS) data and least-squares estimation to determine the system state. Liu et al. [4] first used a discrete wavelet transform to preprocess the ship’s trajectory and then applied grey systems theory to predict the ship’s navigation position. Zhen et al. [5] constructed a three-layer BP neural network in which AIS data from the previous three points were used as the input and the fourth point was given as the output to predict the navigation behavior of the ship. Zhao et al. [6] used an improved Kalman filter algorithm to predict ship trajectories. Tong et al. [7] examined the law of curved navigation and the reliability of ship trajectories and proposed a geographic information system (GIS)-based prediction method for the AIS data of island navigation. Kim and Hong [8] used a nonlinear Kalman filter in a curved-motion interactive multimodel (IMM) algorithm to track vehicles. Mehrotra and Mahapatra [9] applied a four-state Kalman filter and “jerk” model to track predictions via motion models in a three-dimensional (3D) space. Vaidehi et al. [10] used an ordinary Kalman filter with neural network elements and an auxiliary Kalman filtering scheme to track highly maneuverable multi-targets. Gan et al. [11] grouped historical trajectories by the k-means algorithm and then used artificial neural network (ANN) models to predict ship trajectories. Cai and Zhang [12] constructed an offline model using a hybrid particle swarm optimization evolutionary algorithm (PSO-EA) for time series prediction.

Previous online prediction algorithms could not learn or retain the experience of prior data repairs. Examples of online prediction studies include a ship trajectory prediction model based on Gaussian process regression, proposed by Mao et al. [13], in which a 24 min ship trajectory was simulated and predicted by extrapolating from the ship’s existing data. Perera et al. [14] analyzed and tracked multiple ship conditions, combined ship trajectory detection with shipping state estimation, and simulated ship trajectories. Berker et al. [15] applied a two-dimensional (2D) obstacle motion-tracking module to a dynamometer tracking algorithm to improve data quality for navigation purposes. Stubberud and Kramer [16] used a neural-extension Kalman filter to dynamically predict a target state online, thus improving the state estimation capability of existing models. Sang et al. [17] built a prediction model by using change of speed (COS), rate of turn (ROT), speed over ground (SOG), and course over ground (COG) to develop the closest point of approaching (CPA) searching method. Nagai and Watanabe [18] proposed a ship-position prediction model for a path following the ship’s performance on a curved path. Borkowski [19] presented an algorithm of ship-movement trajectory prediction, which considered measurements of the ship’s current position from a number of doubled autonomous devices through data fusion. Zissis et al. [20] employed machine learning and specifically ANNs as tools to add predictive capability to vessel traffic-monitoring information systems (VTMISs). Yin et al. [21] proposed an online sequential extreme learning machine (OS-ELM)-based gray prediction approach for online ship roll prediction. Zhang et al. [22] predicted ship maneuvering in regular waves. Based on a two-time scale model, the total ship motion can be divided into low-frequency maneuvering motion and high-frequency wave-induced motion. Breda [23], in the simulator, created critical vessel traffic scenarios and predicted maneuvering margins of the vessel, indicating the predicted boundaries of safe operation.

A recurrent neural network (RNN) is a deep learning technology designed to deal with time series data [24]. RNNs can achieve high precision for machine learning tasks with time series attribute data. For example, in 2015, Google substantially improved speech recognition in Android phones and other devices by using RNNs. Apple’s iPhone also uses an RNN framework in Siri. Microsoft uses RNNs for speech recognition, virtual-dialogue image generation, and programming code. RNNs have been applied in a wide range of research areas, including translation, document abstraction, speech recognition, image recognition, disease prediction, click-through rate prediction, stock forecasting, synthetic music, and e-commerce fraud detection. In an RNN, feedback is provided from the output variable to the input [25,26]. The feedback variable contains a time delay network. However, training an RNN is complex and convergence cannot be guaranteed. AIS data regarding a ship’s trajectory are given as a time series. In an RNN, increasing the data length may induce a gradient error, or a gradient explosive may occur when error parameters are back-propagated. The gradient explosion phenomenon does not meet the training objectives, and a typical RNN does not provide satisfactory results. Therefore, in a bidirectional RNN structure [27], based on the traditional RNN concept, forward and backward propagation are used to effectively update the weights, thereby increasing the contextual relevance and prediction precision. Instead of a hidden-layer unit, the bidirectional RNN uses a long short-term memory (LSTM) unit. The LSTM unit effectively filters key features, performs selective memory, and solves the problem of RNN processing for long-time data. The LSTM algorithm was proposed by Sak [28] to establish long-term correlations between input values. By replacing the hidden-layer network in an RNN with an LSTM unit [29,30], the problem of gradient dispersion is solved and a new storage unit is created to selectively forget or remember a given operation. In this study, we developed a bidirectional LSTM RNN (BI-LSTM-RNN) combining historical experience and real-time adjustment flexibility that can be utilized to predict the navigation behavior of an unmanned ship during collision avoidance measures against a manned ship. This approach can support a human operator’s better understanding of complex conditions at sea and enhance decision-making to avoid danger.

## 2. Navigation Behavior Learning Model

### 2.1. RNN Structure

**Figure 1:**
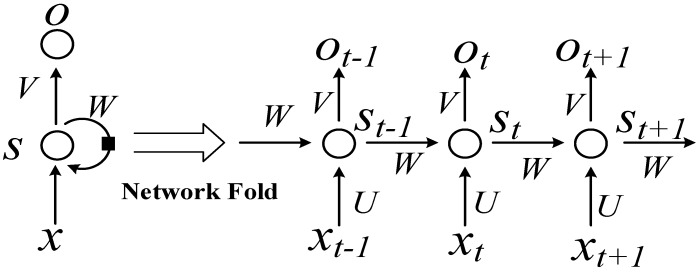
Basic recurrent neural network (RNN) structure.

RNNs use directional loops to address problems in the context of input nodes. The RNNs overcome the connection between a traditional neural network structure layer and the hidden layer. The transition between each layer node is no longer the input of a hidden layer. An RNN is a sequence-to-sequence model [25,26,31] that can suitably process sequence data of any length. In processing AIS data, the current state is related only to the previous ship states. The basic network structure of an RNN is depicted in Figure 1. In the figure, *t* represents the time, *x* is input data, *O* is output data, *S* is the network state, *W* is the update weight, *V* is the weight between the cell and the output, and U is the weight between the input and cell. 

The idea of the RNN structure is to make full use of the information in the previous sequence, which is common in traditional neural networks. It is assumed that all inputs or outputs are independent of each other. However, many natural language processing tasks are judged by context. Therefore, this assumption has its limitations. An RNN is directly translated into a circulating neural network or RNN because different inputs pass through the same neural network, and the difference is concealed by the previous state of the hidden layer. As shown in Figure 1, the unrolled RNN is on the left side and the network structure of the expanded RNN is on the right side.

**Figure 2:**
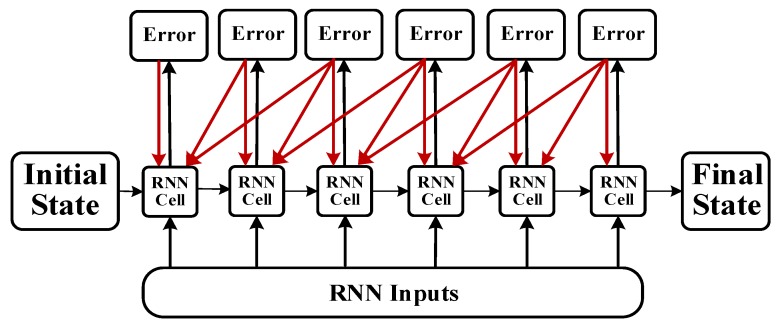
Cell unit interconnection diagram.

An RNN contains input units labeled $\left\{x_{0},x_{1},\ldots ,x_{t},x_{t+1}\right\}$, output units labeled $\left\{y_{0},y_{1},\ldots ,y_{t},y_{t+1}\right\}$, and hidden units labeled $\left\{s_{0},s_{1},\ldots ,s_{t},s_{t+1}\right\}$. The hidden units perform the most important work [32]. A one-way flow of information occurs from the input to the hidden units to the output units (Figure 2). In some cases, the RNN breaks the one-way nature of the second flow, and the boot information is returned to the hidden unit from the output unit. This process is called back-projection, and the input to the hidden layer includes the state of the previous hidden layer. The nodes in the layer can be connected to the next layer or to other units.

### 2.2. LSTM Cell Structure

**Figure 3:**
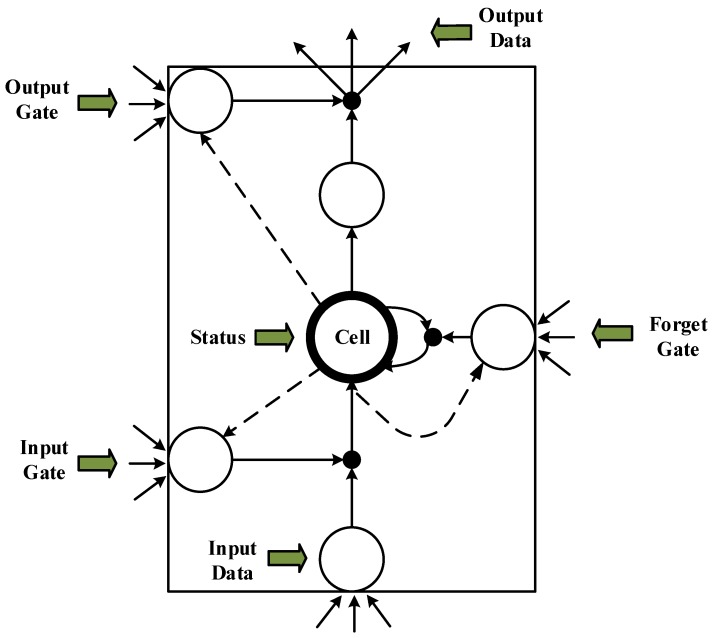
Long short-term memory (LSTM) cell unit structure diagram.

An LSTM unit is a special RNN unit that can solve the long-term dependency problem. In an LSTM unit, the cell state controls the discarding and adding of information through the gate to achieve forgetting and memorizing functions [33,34,35]. An LSTM performs selective operations on the knowledge learned using the three gate structures of input, output, and forget gate. The LSTM structure (Figure 3) allows self-looping and real-time updating of weights to prevent gradient disappearance and gradient expansion.

**Figure 4:**
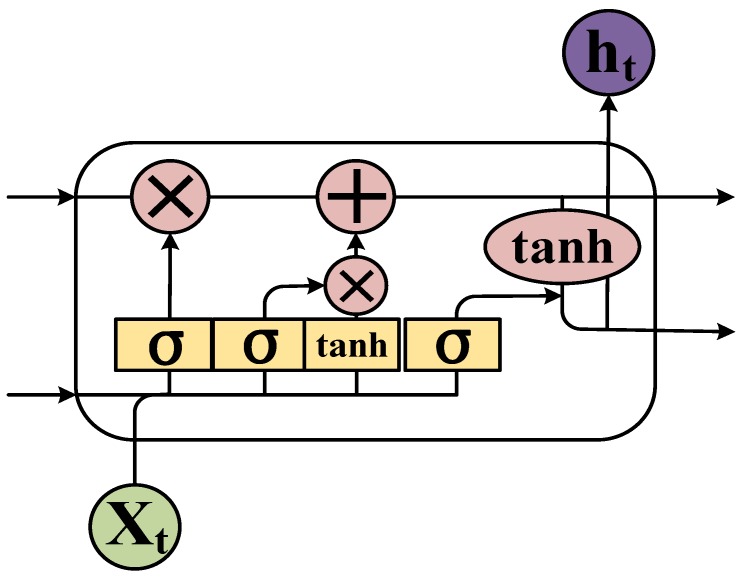
Internal schematic of an LSTM cell unit.

The training process of the LSTM network is as follows:

(1) State initialization: The number of neural nodes in the input layer, number of output nodes, and number of each cell unit (*k*) are determined. The initial state {*S*} of each cell unit is equal to 0, and the link weight ($\omega_{ij}$) of each layer is equal to 0. The average value (±1) is a randomly generated range. The offset $\theta_{j}$ is initialized to 0.1. *W* represents the weight of the matrix.
(2) The output data (*H*) are calculated from the input layer, according to $\omega_{ij}$ and $\theta_{j}$ from Equation (1):
  (1) $${\tilde{H}}_{j}=\sum_{i=1}^{n}\omega_{ij}x_{i}+\theta_{j}.$$
(3) LSTM unit calculation: The output of the unit above the forget gate and the input of this unit are used as inputs for the sigmoid function (Figure 4), which adds to the degree of forgetting used to control the previous unit.
  (2) $$f_{t}=\mathrm{sigmoid}(W_{f}\cdot [h_{t-1},x_{t}]+b_{f})$$
(4) The input gate integrates the *C_t_*_−1_ of the previous state with the *C_t_* of the current state to update the cell unit state.
  (3) $$C_{t}=f_{t}\cdot C_{t-1}+i_{t}\cdot \tilde{C_{t}}$$
  (4) $$C_{t}=\tanh(W_{C}\cdot [h_{t-1},x_{t}]+b_{C})$$
(5) The output value ($o_{t}$) from the output gate is passed to the status value ($h_{t}$) of the next unit to complete the training procedure.
  (5) $$o_{t}=\mathrm{sigmoid}(W_{o}\cdot [h_{t-1},x_{t}]+b_{o})$$
  (6) $$h_{t}=o_{t}\cdot \tanh(C_{t})$$
(6) Error calculation: The prediction error (e) is calculated according to *O* and the expected output (y) of the RNN prediction error, returning the error number of each batch.
  (7) $$e_{k}=\sqrt{y_{k}{}^{2}-o_{k}{}^{2}}\;k=1,2,3\ldots m$$
(7) Weight updating: The random gradient descent method is used to optimize the error depending on the value. For each update parameter, it is unnecessary to traverse all of the training sets; only one value is used to update a parameter. Such an algorithm is more suitable for big data and has fast searching capability.
  (8) $$w_{k}\to w_{k}^{\prime }=w_{k}-\frac{\eta }{m}\sum_{j}\frac{\partial C_{X_{j}}}{\partial w_{k}}$$
  (9) $$\theta_{l}\to \theta_{l}^{\prime }=\theta_{l}-\frac{\eta }{m}\sum_{j}\frac{\partial C_{X_{j}}}{\partial w_{k}}$$

### 2.3. Bidirectional Structure

**Figure 5:**
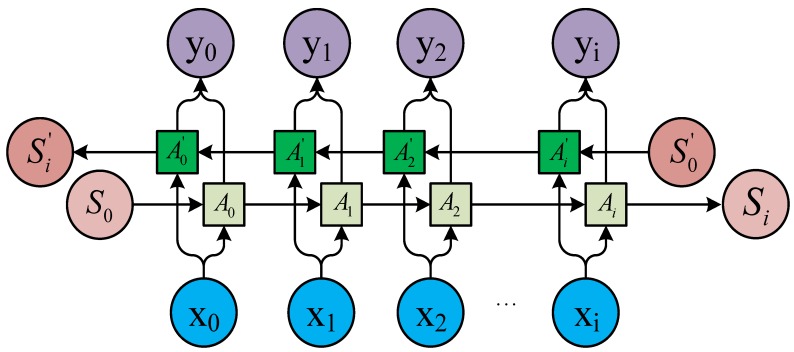
Bidirectional recurrent neural network (BI-RNN) structure diagram.

If the following information can be accessed in advance, the analysis of the current sequence information becomes relatively easy [27,36,37]. As standard RNN processes have time series sequences, the following information is usually ignored. A delay is commonly added between the input and the target to predict future information. However, in practical applications, an excessively long delay yields an inferior prediction result. An inferior prediction result will cause the network to devote excessive resources to memorizing large amounts of input information, thus diminishing its ability to model the combined knowledge of different input vectors. Therefore, the size of the delay must be adapted manually. In a bidirectional RNN, the training sequence involves both forward and backward RNNs, which are connected to an output layer. Figure 5 illustrates the structure of a bidirectional RNN.

The structure of the bidirectional RNN provides complete historical and future information for each point in the input sequence to the output layer. Figure 5 displays the bidirectional RNN spread over time. Six unique weights are used repeatedly in each time step: (*w*1, *w*3) correspond to inputs to the forward and backward hidden layers, (*w*2, *w*5) correspond to hidden layers to the hidden layer itself, and (*w*4, *w*6) correspond to forward and backward hidden layers to the output layer.

**Figure 6:**
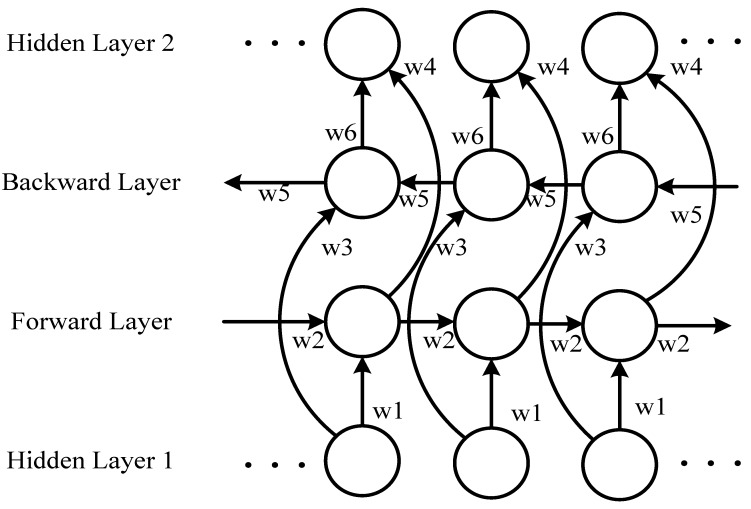
BI-RNN weight relationship diagram.

No information flow occurs between the forward and backward hidden layers, ensuring that the expanded graph is acyclic for the weight relationship (Figure 6).

### 2.4. Batch Training Structure

**Figure 7:**
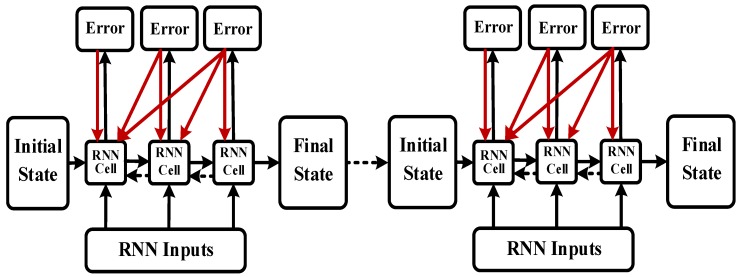
Bidirectional long short-term memory recurrent neural network (BI-LSTM-RNN) batch-training diagram.

In this study, the LSTM unit was added to the time series data to solve the gradient disappearance problem. Moreover, a bidirectional structure was implemented to enhance the context correlation, yielding the BI-LSTM-RNN. Batch training was performed with batches comprising 10 sets of data each. The AIS data were arranged in a matrix and randomly batched, as illustrated in Figure 7, to prevent overfitting, eliminate any self-correlation of the data, and improve the learning efficiency.

## 3. Navigation Behavior Prediction Model

**Figure 8:**
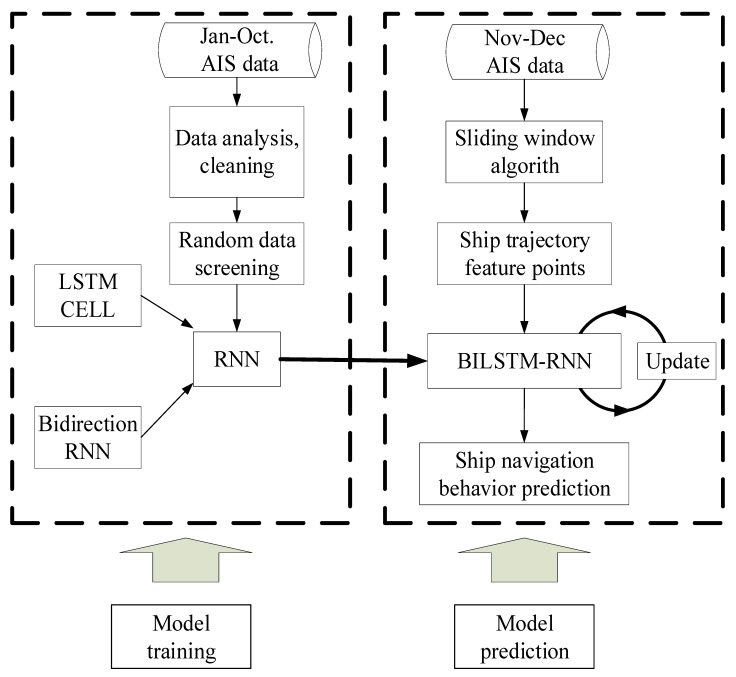
Flowchart of the navigation prediction model using BI-LSTM-RNN.

To verify the validity of the model, 2015 AIS trajectory data from Tianjin Port for 11,032 ships were selected. The data included 36,807,928 coordinate points occupying 8.58 GB. The AIS data from January to October included 29,277,849 points and comprised the training group. The AIS data from November to December included 7,530,103 points and formed the verification group. A flowchart of the complete prediction model is displayed in Figure 8.

### 3.1. Parameter Analysis

**Figure 9:**
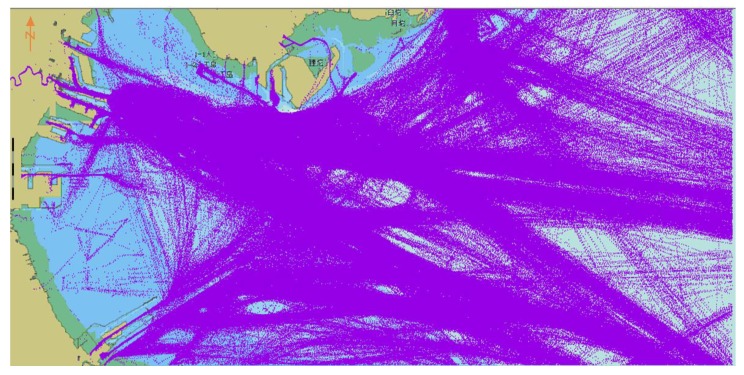
Automatic identification system (AIS) data diagram for the Tianjin Port area in 2015.

For an unmanned ship meeting another ship, it must predict the behavior of the incoming ship and adopt an effective collision avoidance strategy. In our BI-LSTM-RNN, the current and historical AIS ship data are taken as input values, and the future ship position is taken as the output value of the network; the output can then be compared with actual ship position data. The AIS data are multidimensional and multiparametric to characterize ship behavior; for example, the data include the ship’s direction, position, and speed modified over time. In the test AIS data, each ship was subdivided according to its Maritime Mobile Service Identity (MMSI). The ships were sorted using timestamps. The storage structure of the AIS information is displayed in Table 1; the AIS data for June are illustrated in Figure 9.

To avoid overfitting, the position information is comprehensive and includes information regarding speed. Therefore, information related to the position, heading, and time is selected for learning. The input layer ship behavior data can be expressed as

(10) $$\begin{array}{l} I(t)=\{lon_{t-1},lat_{t-1},t-1,heading_{t-1},\\ \;lon_{t},lat_{t},t,heading_{t},\\ \;lon_{t+1},lat_{t+1},t+1,heading_{t+1},\\ \;t+2\} \end{array}$$

Batch standardization is required before data are entered into each layer network:

(11) $$\hat{x_{i}}=\frac{x_{i}-\mu_{B}}{\sqrt{\sigma_{B}^{2}+\varepsilon }}$$

(12) $$y_{i}=\gamma \hat{x_{i}}+\beta$$

where $\mu_{B}$ is the batch mean, $\sigma_{B}^{2}$ is the batch variance, and γ and β are the learned parameters.

The output layer ship position data O(t+2) can be expressed as

(13) $$O(t+2)=\{lon_{t+2},lat_{t+2},heading_{t+2}\}$$

The error function is

(14) $$\begin{array}{l} loss=|heading\_pre-heading_{t+2}|\\ \times \sqrt{{\left(lon\_pre-lon_{t+2}\right)}^{2}+{\left(lat\_pre-lat_{t+2}\right)}^{2}}\\ /sin(\pi -|heading\_pre-heading_{t+2}\left|/2\right) \end{array}$$

**Figure 10:**
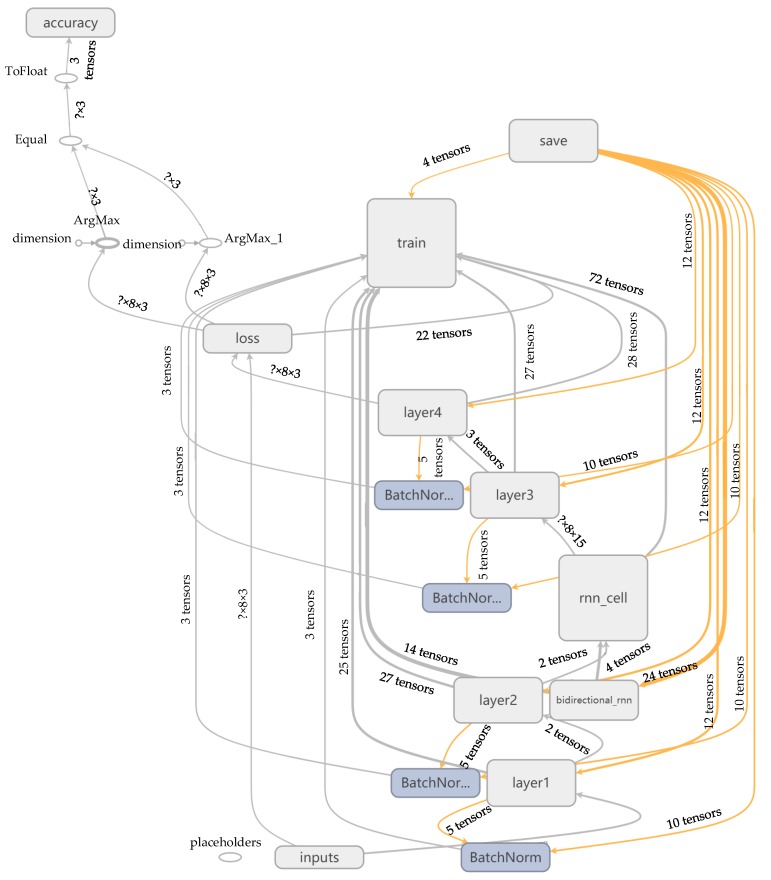
BI-LSTM-RNN structure diagram.

Under the machine learning framework of Google TensorFlow, the BI-LSTM-RNN was implemented in the Python language. The learning network structure contained one input layer, two hidden layers, two LSTM unit layers, and one output layer. The overall structure of the network training, automatically generated through TensorBoard, is depicted in Figure 10.

### 3.2. AIS Trajectory Data Value Filter

**Figure 11:**
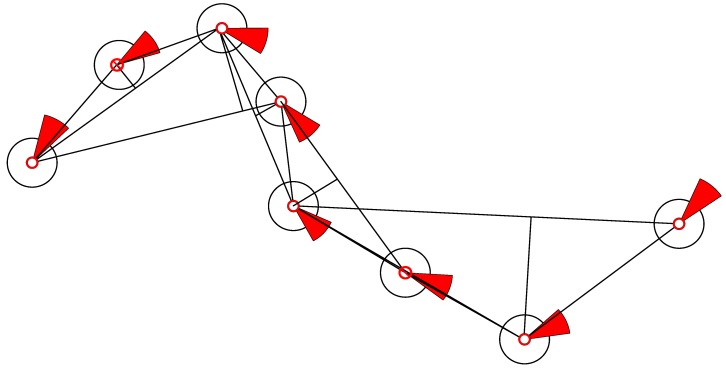
Improved sliding-window compression algorithm.

When the compression algorithm compresses the trajectory data, the algorithm attempts to establish a criterion for judging the value of the ship’s trajectory [38] (Figure 11). It removes data with low trajectory values and retains data with extreme trajectory values. This operation achieves compression and must be retained. Data with high trajectory values are called ship trajectory feature points and key feature points. At feature points, the original trajectory is strong in the AIS ship trajectory data. If such a point is lost, the ability to restore the original trajectory considerably decreases. Points that are not key feature points can be simplified to achieve the compression effect. Eliminating some track data inevitably causes distortion; the threshold is determined to be between the compression and distortion rates.

## 4. Results

**Figure 12:**
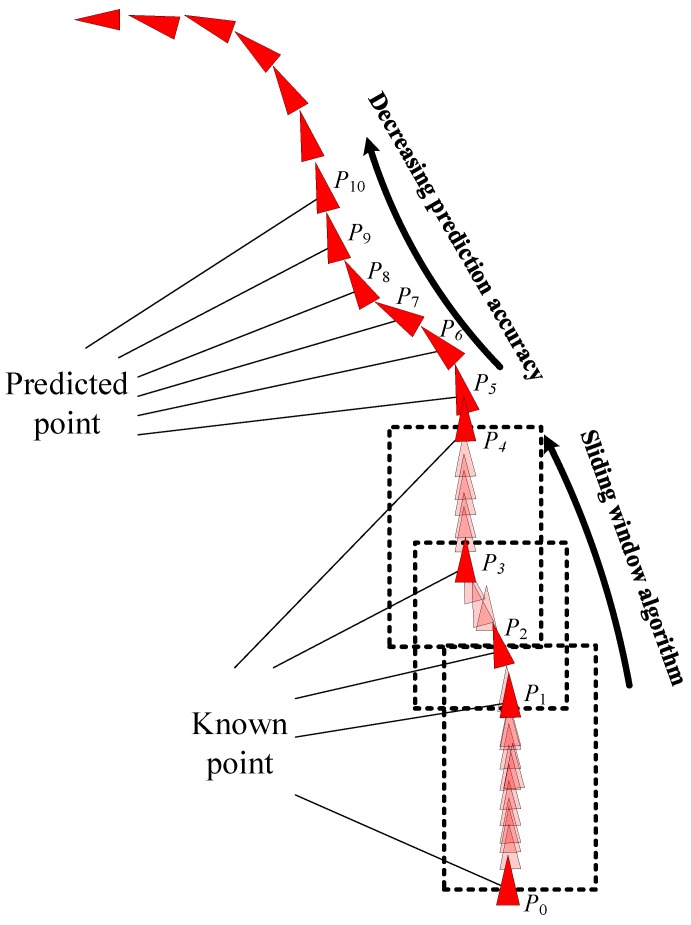
BI-LSTM-RNN trajectory prediction diagram.

Four points from the AIS data were used to form a training sample. The first data point was fixed, and the second, third, and fourth points were intercepted at intervals of three to five points. This selection disrupted the personality association between the data and increased the sensitivity of the data to time parameters. The problems of gradient explosion and gradient disappearance were solved through batch training. The trained network model was highly versatile and directly usable; it did not require retraining for specific areas. The BI-LSTM-RNN network continued to learn online and adjusted the network in the actual application scenario. By using three historical data points at a time, six future ship position points could be predicted. Furthermore, by adjusting the network parameters according to the continuously generated ship data, six new future ship position points could be predicted (Figure 12).

**Figure 13:**
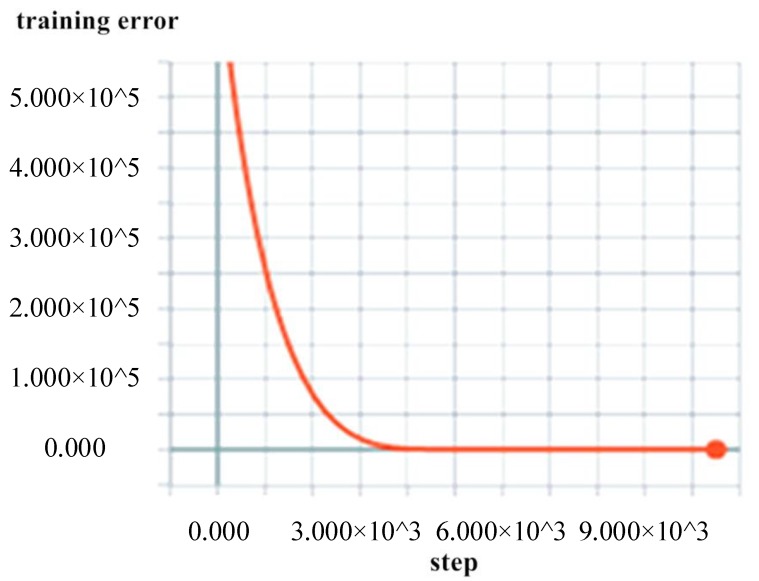
BI-LSTM-RNN training error change diagram.

The data from Tianjin Port for January–October were used as the training group to train the neural network parameters. The error was stable at approximately 90 m. The training error is displayed in Figure 13.

Previously published trajectory prediction algorithms had two major disadvantages: (1) The prediction algorithms mostly included multiple input points and eigenvalues, but only one output point, thus limiting their prediction capacity and consequently restricting their applicability and accessibility in navigation. In this study, the BI-LSTM-RNN structure used three historical ship position data points, and six points could be continuously predicted. Thereafter, the prediction accuracy gradually decreased. (2) Conventional prediction algorithms possess limited versatility, are based on a fixed mathematical model, and are only valid for learning the training data of a ship in advance. Conventional algorithms cannot be adaptively changed, and precise prediction and judgment of the targeted object are impossible, thus the versatility is weak. The BI-LSTM-RNN can be applied to improve versatility by retaining the navigational habits of ships included in AIS big data and using the forget operation on the individual cases of single-ship data. The object is forecasted online, and the characteristics of the current ship can be memorized for a short period of time. Thus, the navigation behavior of a ship can be precisely predicted.

**Figure 14:**
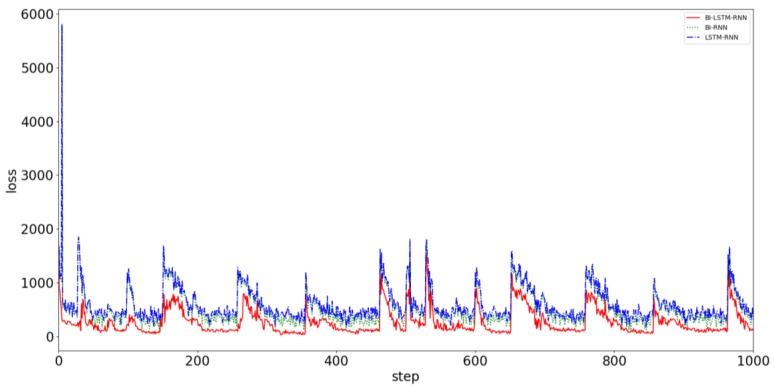
Convergence effects of three prediction models: BI-LSTM-RNN (red line), BI-RNN (green line), and LSTM-RNN (blue line).

Trajectory prediction involves grasping the movement of the ship and obtaining reliable collision avoidance decisions in advance. The trajectory prediction algorithm requires accuracy and timeliness. Greater consistency with reality improves the user’s likelihood of obtaining the correct conclusion. The time available for making collision avoidance decisions is very limited. Therefore, obtaining the prediction result quickly is necessary. To prove the superiority of the proposed prediction algorithm, AIS data from Tianjin Port from November to December, which included 7,530,103 data points, were used as the verification group. The data were not intercepted when the network was being trained, and all the data were directly taken as input for the navigation behavior of the ship. The ship used the BI-LSTM-RNN, BI-RNN, and LSTM-RNN to compare prediction errors. Figure 14 displays a comparison of the convergence effects of the three algorithms.

**Figure 15:**
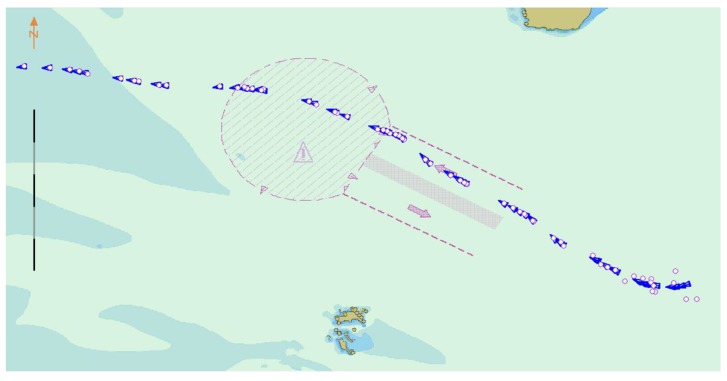
Schematic of ship behavior prediction.

**Figure 16:**
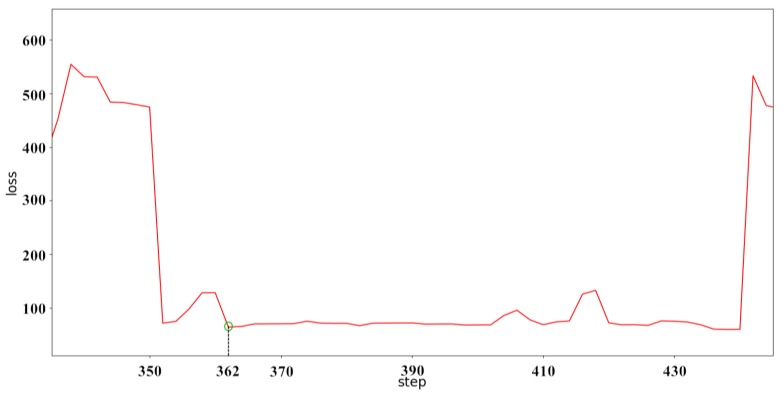
Single-ship prediction error diagram for verification group.

It can be clearly seen from Figure 14 that the neural network predicts the behavior of the ship. When predicted for a period of time, the data of a single ship in the verification dataset are limited, and the data after verification are replaced with the data of the other ship to continue the prediction and verification. Although the prediction accuracy deteriorates suddenly, it always converges in a short time and stabilizes. The convergence velocity, oscillation amplitude, and prediction accuracy of the BI-LSTM-RNN are superior to those of the LSTM-RNN and bidirectional RNN. The experimental results indicate that the BI-LSTM-RNN is trained to predict the position of a single ship in a short time, as illustrated in Figure 15 and Figure 16.

It can be seen from Figure 16 that although each conversion of the new ship data image will cause the rebound phenomenon to occur, it can quickly converge and stabilize in a short time. After about 15 batches of training, the predictive performance of the BI-LSTM-RNN appeared to be stable. The reliability of the navigation behavior prediction was 10 m or less within the accuracy of GPS positioning. The BI-LSTM-RNN was then applied as a prediction module. 

## 5. Conclusions

We inferred the following from our experiment:

1. Selecting an RNN for the time-series data characteristics of AIS big data allows training regarding the general rules of ship maneuvering and motion characteristics.
2. Adding the LSTM unit improves the gradient loss caused by infinite-sequence data in the loop training. An RNN can remember the common features of the AIS big data and forget personality differences. Thus, the RNN has an autonomous choice to remember or forget.
3. By incorporating a two-way RNN structure, the network can learn the information provided by historical data and optimize the network by using future data. The current prediction can establish a strong correlation related to the context.
4. The trained BI-LSTM-RNN can accurately predict future ship navigation behavior and adjust parameters in real time with existing data as input.

The experimental results proved the reliability of the model. Trajectory prediction with the BI-LSTM-RNN can provide security for navigation and assist in trajectory planning and risk monitoring. This research provides a theoretical basis for the design of innovative intelligent collision avoidance systems for unmanned ships, prediction of the navigation behavior of other ships, and mitigation of the risks of vessel traffic service (VTS) management. This study also provides a theoretical basis for subsequent research. The BI-LSTM-RNN is suitable for shipment tracking, ship classification, and ship identification. Moreover, the determined vessel state can be used to estimate the future navigation trajectory, which ultimately assists in predicting the future sailing behavior and maneuvering intentions of a ship and generating an accurate collision avoidance strategy.

## Figures and Tables

**Table 1:** Automatic identification system (AIS) data structure storage. MMSI, Maritime Mobile Service Identity.

MMSI	Heading (°)	Course (°)	Speed (Kn)	UnixTime (ms)	Lon_d (°)	Lat_d (°)
100900000	5.0	5.6	2.4	1,422,721,505	117.997973	38.669028
100900000	2.0	2.5	2.4	1,422,721,943	117.998265	38.673763
100900000	208.0	208.1	2.9	1,422,723,323	117.999900	38.677282
100900000	177.0	177.3	2.7	1,422,728,073	118.000740	38.682742
100900000	183.0	183.8	2.7	1,422,753,718	118.011620	38.683072
100900000	185.0	185.4	2.6	1,422,814,193	117.996735	38.675307
100900000	216.0	216.4	2.9	1,422,820,375	118.000242	38.684793
100900000	184.0	241.5	3.4	1,422,845,041	117.993773	38.679000
100900000	187.0	184.6	2.5	1,422,851,329	117.991593	38.677032
100900000	180.0	187.7	2.8	1,422,925,499	117.991617	38.654512
